# Impact of Noodle Formulation, Boiling Methodology and Their Interactions on Stable Hydrogen and Oxygen Isotope Ratios

**DOI:** 10.3390/foods13060959

**Published:** 2024-03-21

**Authors:** Jingjie Yang, Sara Wilhelmina Erasmus, Qianqian Sun, Boli Guo, Saskia Marieke van Ruth

**Affiliations:** 1 Comprehensive Utilization Laboratory of Cereal and Oil Processing, Institute of Food Science and Technology, Chinese Academy of Agriculture Sciences, Ministry of Agriculture and Rural Affairs of the People Republic of China, Beijing 100193, China; jingjie.yang@wur.nl (J.Y.); sunqianqian@caas.cn (Q.S.); 2 Food Quality and Design Group, Wageningen University and Research, P.O. Box 17, 6700 AA Wageningen, The Netherlands; sara.erasmus@wur.nl; 3 School of Agriculture and Food Science, University College Dublin, D04V1W8 Dublin, Ireland

**Keywords:** boiling processes, extruded noodles, gluten/starch formulation, δ^2^H, δ^18^O

## Abstract

Stable isotopes are commonly utilized for the geographical origin verification of foods, including wheat. However, assessing processed products poses a greater challenge due to the alterations that take place during processing and which have not been fully elucidated yet. In the current study, the effects of the formulation (the mass ratios of gluten to starch), boiling process and their interaction on the stable hydrogen (δ^2^H) and oxygen (δ^18^O) isotopic ratios of wheat noodles were evaluated. The δ^2^H and δ^18^O of noodles with different formulations (the mass ratios of gluten to starch) as raw materials, in uncooked and cooked (boiled in water) noodles, were examined. The results indicated that the δ^2^H of the boiled noodles ranged from −80.1‰ to −46.8‰ and were significantly lower than those of the raw materials, which ranged from −73.0‰ to −39.2‰, and the uncooked noodles, which ranged from −73.3‰ to −39.6‰. Oppositely, ^18^O was enriched in the boiled noodles, ranging from 27.7‰ to 31.3‰, compared with the uncooked noodles, ranging from 28.4‰ to 29.6‰. In addition, a significant interaction effect between the formulation and the boiling process was recorded for δ^18^O. This study shows that the hydrogen and oxygen stable isotopic compositions of noodles were significantly changed during the boiling process, and the isotopic fractionation varies with the different formulations.

## 1. Introduction

Food traceability is the ability to follow the movement of a food product and its ingredients through all steps in the supply chain, both backward and forward. Traceability involves documenting and linking the production, processing and distribution chains of food products and ingredients. The identification of the geographical origin of foods is one of the most important issues in the field of food traceability. The geographical origin adds value to certain products, and it is also used as an identifier of food safety risks. Geographical-origin-labeled foods are generally associated with a high level of quality and safety and usually charge a premium price. However, these premium foods may be replaced by inferior or counterfeit products for financial interests, which harms both consumers and legitimate producers. Wheat (*Triticum aestivum* L.) is the most important cereal crop and is consumed as a staple food around the world, providing nutrition and energy to half of the world’s population [1,2]. The high consumption of wheat and its processed products and the desire of consumers to know the geographical origin of their food and whether it complies with food labeling have resulted in a need for a method to identify the geographical origin of wheat and wheat products [3].

With the advancement of analytical technology, methods including stable isotope and multi-element analyses, liquid and gas chromatographic techniques, molecular biology methods and spectroscopic techniques have been applied in favor of food traceability over the last few decades [4]. Among them, stable isotope analysis (SIA) has become a common and relatively efficient analytical method for determining the geographical origins of foods. The elements with the most used isotopes include carbon, hydrogen, oxygen, nitrogen and sulfur, which are very stable and do not decay through radioactive processes [5]. Stable isotope analysis has been frequently used for verifying the geographical origin of wheat kernels, such as identifying wheat from different countries or different provinces of China [6,7] and distinguishing organically grown wheat from conventionally farmed wheat [8,9]. Meanwhile, the influence of some factors, such as variety, genotype, harvest year and their interaction effects, on the stable isotopes used for the geographical traceability of wheat has also been reported [10]. To further apply isotopic analysis to processed products, it is necessary to evaluate and understand the changes in and conversion of stable isotope ratios during processing. In recent years, some experts have focused on stable isotope variations in wheat products along the production chain, like wheat flour [11,12], bread [13], pasta [14] and Chinese dried noodles [15]. The cooking process is also an essential procedure before food is ready to eat. For wheat-processed or heating ready-to-eat products, the issues about whether the cooking process would alter stable isotopic ratios in such processed products and whether the variation in stable isotopic ratios in such products during processing will influence the results of their raw materials’ geographical origin identification are still not fully elucidated.

Previous studies have reported that the cooking process may alter the original stable isotopic ratios in other food types. For instance, Fernandes et al. [16] investigated the influence of boiling, grilling and steaming on the stable isotope ratios of raw flesh and their fractionations and reported that the cooking process changed the composition of some fractions, but the changes in isotopic ratios relative to the raw materials were in general < 1‰. Similarly, Zhou et al. [17] studied the effect of the different beef processing operations (boiling, frying and roasting) on the stable C, N and H isotopic compositions, indicating that there was a significant difference in δ^2^H between raw beef and processed beef products but not in the δ^13^C and δ^15^N isotope ratios. Moreover, Royer et al. [18] indicated that the cooking process, including boiling, roasting and frying, significantly changed δ^18^O in animal meat products but did not affect the δ^13^C and δ^15^N isotope ratios. The studies above all relate to animal-based products. The effect of processing on stable isotope ratios in plant-based products has not received much attention yet, which includes the influence of the cooking process on the δ^2^H and δ^18^O of wheat-processed products.

Boiling is one of the most important cooking methods for noodles, in which the presence of water is essential. Previous studies proposed that the addition and evaporation of water during the production chain would influence the hydrogen or oxygen isotopic composition of the final pasta noodles [14,19]. But it lacks the support of evidence. In addition, gluten and starch are the main components of processed wheat products. They have different water absorption capacities and water-binding capacities. During the manufacture of noodles and the boiling process, noodles produced by different formulations (the mass ratios of gluten to starch) will combine and interact with the moisture to different degrees. Therefore, during the boiling process, the variation in δ^2^H and δ^18^O in different wheat products might be varied. However, fewer studies have focused on it. All the issues mentioned above have limited the application of isotope analysis on the traceability and authentication (i.e., determining the geographical origin) of wheat-containing products.

In the current study, different formulations were produced by varying the proportions of wheat gluten and wheat starch powder to form noodle samples with different water absorption and holding capacities. Following this, noodles were manufactured and subsequently cooked in water. The effects of the ingredients and processing on δ^2^H and δ^18^O in the raw materials, their semi-products (uncooked noodles) and their heating-to-eat products (cooked noodles) were elucidated. This study will help to understand how the cooking process alters hydrogen and oxygen stable isotopic ratios in different processed wheat-containing products. Furthermore, it will provide a theoretical basis for applying valid analytical methods for the traceability and authentication of processed wheat products in future studies.

## 2. Materials and Methods

### 2.1. Raw Materials Used for Noodle Production

The same batches of wheat gluten powder (100%) and pure starch powder (100%) were bought from Xinrui Group Co., Ltd., Liaocheng, Shandong Province, China. 

### 2.2. Experimental Procedures

The experimental process included mixing gluten and starch, mixing with water, extruding and cooking. All the experiments were performed in triplicate.

#### 2.2.1. Raw Material Flour Preparation

The total weight of every flour sample was 1000 g, and gluten and wheat starch were weighted based on eight formulations of gluten powder and wheat starch powder (0:100, 15:85, 30:70, 45:55, 55:45, 70:30, 85:15 and 100:0 (*w*/*w*)). The blended flour samples were obtained by constantly hand shaking in a sealed bag for 3 min. The mixing step was performed in triplicate for each formulation.

#### 2.2.2. Manufacturing of Fresh Extruded Noodles

Deionized water (δ^2^H = −75.8‰, δ^18^O = −10.3‰) was added to the eight raw materials and the moisture content of the mixture was adjusted to 35% (*w*/*w*). Then, the mixture was pulled into the pilot-scale, corotating and intermeshing twin-screw extruder (DSE-25, Brabender, Duisburg, Germany). The working parameters were set as follows: 25 mm screw diameter, 20:1 screw length/diameter ratio, a circular die with a 3 mm diameter and working at room temperature. The extruded noodles were collected after the extruder reached a steady state. Then, the fresh noodles were cut into lengths of 20 cm on a glass plate immediately. Then, 100 g of fresh noodles were cut into 2 cm threads and subsequently freeze-dried for 72 h before being ground to a fine powder using a ZMM400 mill (Retsch, Haan, Germany), after which a stable isotopic ratio analysis was performed on the powdered samples. All powder samples were sieved through a 100-mesh screen. The rest of the fresh noodles were packaged into a polyethylene bag and stored in a freezer at −18 °C until boiling. 

#### 2.2.3. Boiling of Extruded Noodles

Before boiling, the 100 g frozen noodle samples were weighed and thawed for 60 min at room temperature. Then, the 20 cm-in-length noodle samples (100 g) were placed in a 22 cm diameter stainless steel pot with 1000 mL of boiling deionized water (δ^2^H = −75.8‰, δ^18^O = −10.3‰). The pot was then heated on an induction cooker at 1500 W. The cooking process was conducted according to the noodles’ optimum cooking time [15]. The optimum cooking time was measured according to AACC Approved Method 66-50.01 [20]. Specifically, a noodle string was collected every 30 s and pressed between a pair of transparent glass slides to check for the presence of whiteness and a hard core in the noodles. The time when the whiteness and hard core of the noodles disappeared after staying in the boiling cooking water was regarded as the optimum cooking time for the noodles [21]. The optimal cooking times of each noodle are presented in Table A1 (provided as Appendix A). After that, the noodles were completely cooked, as described in Section 2.2.2.

### 2.3. Stable Isotope Determination

The δ^2^H of non-exchangeable hydrogen as well as δ^18^O in the samples were measured using a comparative equilibration approach in which the samples were equilibrated and analyzed alongside international standards (USGS54, USGS55 and KHS keratin for δ^2^H and KHS keratin and IAEA-601 for δ^18^O). The samples and reference materials were freeze-dried with a vacuum freezer dryer (CHRIST, Osterode, Germany) under −50 °C and 0.1 mbar for 72 h to remove all exchangeable water. Subsequently, the dried powders (1 ± 0.2 mg) were weighed into silver capsules (6 mm × 4 mm), which were folded and compressed to contain the sample and to remove any air. Then, the encapsulated samples alongside the standard materials were equilibrated in laboratory atmospheric conditions for at least 5 days before measurements [22]. The approach follows the recommended Principle of Identical Treatment (PIT) for stable isotope analyses. The measurement was carried with an Isotope Ratio Mass Spectrometry (IRMS) system (IsoPrime100, Isoprime, Manchester, UK). The detailed working parameters were as follows: the cracking temperature: 1450 °C, the flow rate of the carrier gas (helium): 120 mL/min, the reference gas pressure: 1200 mbar and the TCD flow rate: 120 mL/min. The stable isotope ratios are expressed in the delta (δ) notation and calculated against the Vienna Standard Mean Ocean Water (V-SMOW), which was calculated based on Equation (1):

δ (‰) = ((R_sample_/(R_standard_−1)) × 1000 (1)

where δ (‰) represents the value of δ^2^H or δ^18^O, whereas R is the ratio of ^2^H/^1^H or ^18^O/^16^O. 

δ^2^H and δ^18^O were calculated against reference substances USGS54 (Canadian lodgepole pine), USGS55 (Mexican ziricote), KHS keratin (Kudu horn) and IAEA-601, respectively. These standards were used because they are the most matrix-matched references to the noodle samples used in this study. The δ^2^H and δ^18^O of the noodle samples are reported relative to the VSMOW-SLAP scale, and on this scale, the δ^2^H of non-exchangeable USGS54, USGS55 and KHS keratin is –150.4 ± 1.1‰, –28.2 ± 1.7‰ and −35.3 ± 1.1‰, respectively, and the δ^18^O of KHS keratin and IAEA-601 is +21.2 ± 0.17‰, +23.3 ± 0.3‰, respectively. Samples were analyzed in triplicate, and the average of the three was taken. In total, the number of measured samples (N) was 72 (8 formulations × 3 mixing replicates × 3 (1 flour; 2 noodle types)). 

The analytical precision of the laboratory reference was smaller than 1‰ in δ^2^H and 0.2‰ in δ^18^O when measuring the three replicates.

### 2.4. Statistical Analysis

The stable isotope ratio data were first tested for a normal distribution using the Shapiro–Wilk test [23]. After confirmation of normality, a one-way analysis of variance (ANOVA) followed by Duncan’s multiple range test was applied to determine the statistically significant difference with a 5% probability level [24]. Scatter plots were created to show the difference in stable isotope ratios among the raw materials, uncooked noodles and cooked noodles. A combined analysis of variance was conducted to assess the formulation, the boiling process and their interaction effects on H and O isotopes using general linear models (GLM) by computing the contribution rate following the mean square value. All the statistical analyses were performed using SPSS 22.0 software (SPSS Inc., Chicago, IL, USA) and Origin 2021b (Origin Lab Corporation, Northampton, MA, USA). 

## 3. Results and Discussion

### 3.1. The Effects of the Formulation and Boiling Process on the Stable Hydrogen Isotope Ratios in Noodles

#### 3.1.1. Effects of Formulation

Table 1 shows the mean and standard deviation of δ^2^H in the samples with different formulations, i.e., wheat gluten/starch proportions. The results showed that the value of δ^2^H significantly decreased with an increase in gluten content from 0% to 100% in the raw materials, uncooked noodles and cooked noodles (*p <* 0.05), respectively. A previous study showed that the order of relative global ^2^H content can be established as follows: carbohydrates > bulk material > proteins [25]. In the present study, the pure gluten powder (protein) was significantly depleted in ^2^H as compared to the pure starch (carbohydrates), confirming this report. Additionally, the fact that the starch was enriched in ^2^H might be related to the isotopic fractionation involved in the pathway that led to its biosynthesis. In photoheterotrophic conditions, high starch contents in plant leaves indicate that a significant amount of exogenously supplied sugar has been metabolized to triose phosphate (TP). These compounds pave the way to the chloroplasts, the site of starch synthesis, where a proportion is involved in various steps of reductive pentose phosphate (RPP) pathways, together with the release of hydrogen by NADPH, before either existing in the chloroplasts or being transformed to glucose and polymerized to starch. Numerous steps along this path let hydrogen exchange with water, which is responsible for the enrichment of ^2^H in starch [26].

#### 3.1.2. Comparison of Raw Materials, Uncooked Noodles and Cooked Noodles

**Figure 1:**
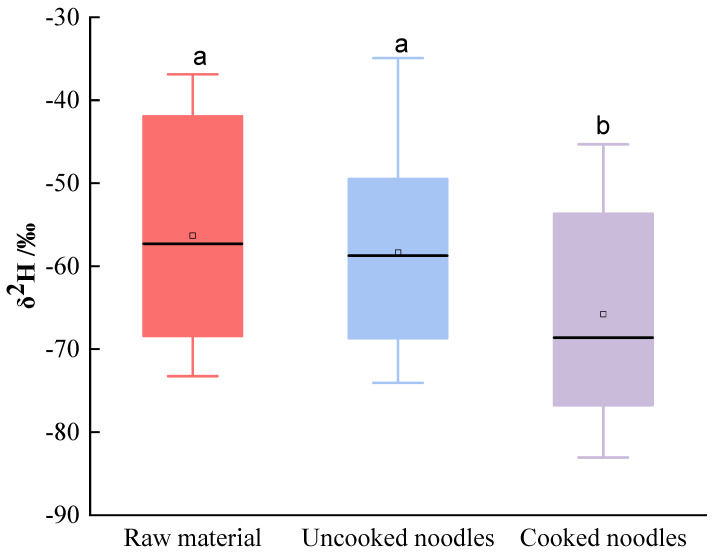
Characterization of the δ^2^H of non-exchangeable hydrogen in the raw materials, uncooked noodles and cooked noodles. (a, b represent significant differences (*p* < 0.05) among different products according to Duncan’s multiple range test).

δ^2^H showed a decreasing trend from the raw materials to the uncooked noodles and subsequently to the cooked noodles; in particular, significant differences can be observed between the uncooked noodles and the boiled noodles (*p <* 0.05) (Figure 1), indicating that the cooked noodles were depleted in ^2^H. However, no significant difference was observed between the raw material and uncooked noodles in terms of δ^2^H, which indicates that the fractionation effect of stable hydrogen isotopes occurred during the cooking process. Wadood et al. [15] also suggested that the hydrogen isotopic ratios of Chinese dried noodles are affected by the boiling process. Additionally, Zhou et al. [17] proposed that the boiling process significantly increases the δ^2^H of beef. A series of physicochemical variations, such as protein transitions and starch gelatinization, occur during boiling, followed by the disruption of non-covalent bonds, which both likely lead to a depletion in ^2^H. Heating could also trigger protein unfolding through a shift in hydrogen bonds and protein re-aggregation through hydrophobic interactions and disulfide bonds, as well as the re-arrangement of new secondary structures of the protein [27,28]. Wang et al. [29] showed that the thermal treatment of gluten could induce a decline in protein surface hydrophobicity, suggesting that more hydrophobic groups will be buried on the surface. As a result, more cooking water will enter the noodles and combine with the hydrophilic groups on the protein surface. Another important reason may be linked to H isotopes’ exchange between organic matter in noodles and exogenous cooking water. In the present study, the noodle samples were composed of wheat gluten and starch powder, which include hydrogen (-H) atoms bound to carbon (-CH) or bound within carboxyl (-OH), amide (-NH) or minimal sulfhydryl (-SH) side groups [30]. The carbon-bound hydrogen isotopes are non-exchangeable with exogenous water; however, the H atoms in the side groups will exchange with hydrogen atoms from the cooking water. The δ^2^H of the cooking water was −75.8‰, which was lower than that of the uncooked noodles. Thus, the heavier isotopes (^2^H) in noodles may exchange with lighter isotopes (^1^H) in the cooking water, resulting in a decrease in the δ^2^H of the cooked noodles. 

Additionally, the average fractionation value of δ^2^H from the raw material to the uncooked noodles was −2.0‰, with this value ranging from −5.5‰ to −0.2‰. Furthermore, the average fractionation value of δ^2^H from the uncooked noodles to the cooked noodles was –7.4‰, with this value ranging from –11.3‰ to –6.9‰ (Table 2). The differences in fractionation values are likely to result from formulation differences. Specifically, the water absorption capacity of pure starch and pure gluten differed significantly, resulting in variation between the water-binding capacity of the raw materials and the differently formulated noodles. Hence, the water absorption and the degree of external water (δ^2^H = −75.8‰) combined with the noodles differed as well. More research is required to confirm the mechanisms behind the differences observed. 

### 3.2. The Effects of the Formulation and Boiling Process on the Stable Oxygen Isotope Ratios in Noodles

#### 3.2.1. Effects of Formulation

The mean value of δ^18^O ranged from 29.1 to 31.5‰, 28.4 to 29.6‰ and 27.7 to 31.3‰ in the raw materials, uncooked noodles and cooked noodles, respectively (Table 3). The cooked noodles with different formulations of gluten and starch showed a significant difference; however, no notable difference was observed in the different raw material flour formulations (except for the raw material composed of 0% and 15% gluten powder) and different uncooked noodles. The results indicate that the ingredients only had a slight impact on the δ^18^O of the raw materials and uncooked noodles. Subsequently, the difference in the cooked noodle products with different formulations could be attributed to the boiling process.

#### 3.2.2. Comparison of Raw Materials, Uncooked Noodles and Cooked Noodles

**Figure 2:**
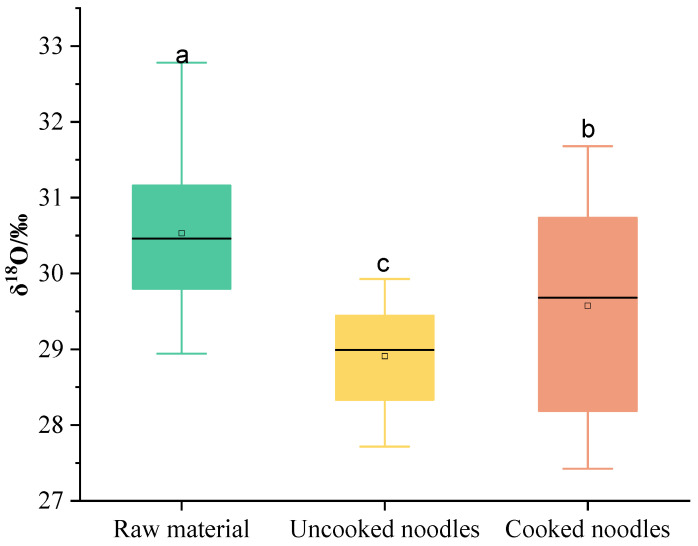
Characterization of δ^18^O in the raw materials, uncooked noodles and cooked noodles. (a–c represent significant differences (*p* < 0.05) among different products according to Duncan’s multiple range test).

Overall, variability in the mean δ^18^O can be seen in the raw materials, uncooked noodles and cooked noodles (Figure 2). Specifically, the raw materials had the highest mean value of δ^18^O, whereas the uncooked noodles had the lowest (*p <* 0.05). The decrease in the mean value of δ^18^O could be attributed to the added water (δ^18^O = −10.3 ‰) during extrusion. Moreover, after boiling, the mean value of δ^18^O increased considerably, demonstrating that the boiling process altered δ^18^O in the noodles. The average fractionation value of δ^18^O from the raw material to the uncooked noodles was −1.5‰ and varied in a range of −2.5‰ to −0.7‰. Additionally, the fractionation value of δ^18^O from the uncooked noodles to the cooked noodles was in the range of −0.7‰ to 1.7‰, and the average was 0.7‰ (Table 4).

Compared to other reports, the present study shows new findings in the case of O isotopic changes during the cooking process. Yoshida et al. [31] pointed out that δ^18^O in kabocha pumpkin was not altered after boiling in water. One reason for these differences may the differences in the properties of the raw materials and the processing conditions. In this study, the noodle samples were cooked in boiling water (100 °C) directly. Andrus et al. [32] suggested that boiling at a high temperature or directly burning the foods on fire for a long period can alter δ^18^O due to the isotopic exchange occurring between H_2_O and organic substances. Another reason could be attributed to the type and composition of the products. The materials used in this experiment comprised different formulations of gluten and starch, which have various physical–chemical properties, especially in terms of water absorption and water-binding capacity. During boiling, the water absorption rate of noodles could be up to 160%. This suggests that the noodles would absorb more water, and thus more boiling water had a better chance to interact with the macromolecular components in the noodles or exchange isotopes with the organic substance compared with otoliths and kabocha pumpkin. Wadood et al. [15] found that δ^18^O increased very slightly in the cooked noodles compared with the dried noodles. However, the enrichment phenomenon was not significant, like in the authors’ present study. The two studies worked with different noodle formulations and water with different δ^18^O, which may have affected the results.

It is also interesting that the fractionation values of the H and O isotopes are different among all the groups. From the raw materials to the uncooked noodles and further to the cooked noodles, water is one of the most important components, which is also highly related to the hydrogen and oxygen isotopic composition. In this experiment, we first used raw materials that have different physical–chemical properties, especially in terms of water absorption and water binding capacity. Previous studies have confirmed that gluten and starch have different capacities in terms of binding or entrapping water [33,34]. Furthermore, during boiling, the water that enters the noodles will exist as free water first, and then will turn into bound water, which closely interacts with macromolecules [35]. That means that the population and distribution of water states varied in the different noodle samples. Thus, the formulation of gluten and starch in the material depends on the content of cooking water absorbed in the noodles during processing. Following this, exchanges between the noodles’ inner water and the exogenous cooking water will occur to different degrees, resulting in varied fractionation values. However, the exact exchange mechanism and the relationship between the change in water status and isotopic ratios need to be elucidated in the future.

### 3.3. Multiway ANOVA

**Figure A1:**
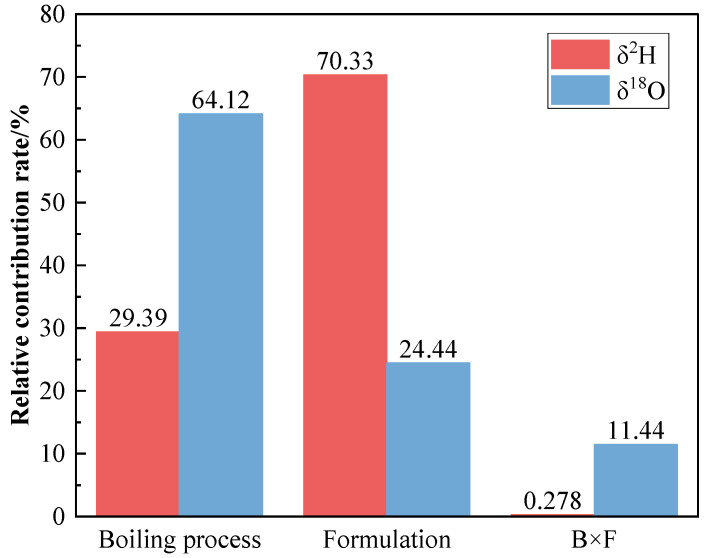
The percentage of the total square variance of each factor for δ^2^H and δ^18^O in the noodles.

A combined analysis of variance was performed to assess if the processing and ingredients of the noodles and their interaction had an impact on the H and O isotopes using a general linear model (GLM) (Table 5). Generally, the mean square (MS) value was used to evaluate the relative contribution rate for all the factors (Figure A1). A high mean value suggests the highest contribution rate. For δ^2^H, the contribution was in the following order: formulation > boiling process > formulation × boiling process. Regarding δ^18^O, the highest contribution rate could be attributed to the factor boiling process followed by the factor formulation, and the lowest contribution was also observed for the interaction of the factors formulation × boiling process. Overall, the formulation and boiling process were the factors that contributed most to the differences in the stable isotope ratios observed. 

## 4. Conclusions

In this study, the potential impact of the formulation and boiling process on δ^2^H and δ^18^O in wheat noodles was evaluated. The results show that both the formulation and boiling processes affect δ^2^H and δ^18^O significantly. In addition, the formulation is the main factor which influenced δ^2^H, followed by the boiling process and the formulation × boiling process interaction. Regarding δ^18^O, the trend was in the order of boiling process > formulation > formulation × boiling process. The formulation significantly influenced δ^2^H in the raw materials, uncooked noodles and cooked noodles. However, for δ^18^O, only a slight impact of the formulation can be observed in the raw material and uncooked noodles. The boiling process had an enrichment effect on the isotopic composition of oxygen and a depletion effect on the isotopic composition of hydrogen. The fractionation values differed in the noodles with different gluten and starch contents. It is proposed that the various fractionation values likely relate to the noodles’ capacity to bind water and the interaction, as well as the exchange between cooking water molecules and the inner macromolecules of the noodles.

The current study will provide a theoretical basis and data reference for applying valid analytical methods for the traceability and authentication of processed wheat products in food supply chains in the future. This will help to ensure fair competition across supply chains and underpin consumer confidence.

Future work should focus on the relationship between changes in water status and distribution and isotopic fractionation values in noodles with different formulations of gluten and starch. Meanwhile, the hydrogen and oxygen variation mechanisms during processing also deserve attention in future research.

## Figures and Tables

**Table 1:** The mean ± standard deviation of the δ^2^H of non-exchangeable hydrogen in the raw materials, uncooked noodles and cooked noodles with different formulations of wheat gluten powder and starch powder.

Gluten–Starch Ratio [*w*/*w*]	Raw Materials	Uncooked Noodles	Cooked Noodles
0:100	−39.2 ^aA^ ± 0.0	−39.6 ^aA^ ± 0.8	−46.8 ^aB^ ± 1.2
15:85	−41.1 ^aA^ ± 1.4	−46.6 ^bB^ ± 2.1	−51.4 ^bC^ ± 0.1
30:70	−48.3 ^bA^ ± 5.0	−51.4 ^cA^ ± 3.2	−56.6 ^cA^ ± 0.1
45:55	−54.1 ^cA^ ± 0.0	−56.3 ^dA^ ± 0.8	−64.4 ^dB^ ± 1.0
55:45	−60.1 ^dA^ ± 1.4	−62.2 ^eA^ ± 0.3	−73.6 ^eB^ ± 1.1
70:30	−66.0 ^eA^ ± 1.0	−67.8 ^fgA^ ± 0.4	−75.8 ^efB^ ± 1.2
85:15	−69.6 ^efA^ ± 0.3	−69.8 ^fA^ ± 0.2	−77.6 ^fgB^ ± 0.3
100:0	−73.0 ^fA^ ± 0.1	−73.3 ^gA^ ± 0.5	−80.1 ^gB^ ± 2.3

Note: in a–g, different mean values in the same column with superscripts of lowercase letters are significantly different (*p <* 0.05) according to Duncan’s multiple range test. In A–C, different mean values in the same line with different superscripts of uppercase letters are significantly different (*p <* 0.05) according to Duncan’s multiple range test.

**Table 2:** The **∆**δ^2^H value ofraw materials, uncooked noodles and cooked noodles with different formulations of wheat gluten powder and starch powder.

Gluten–Starch Ratio [*w*/*w*]	∆δ^2^H_uncooked–raw_	∆δ^2^H_cooked–raw_	∆δ^2^H_cooked–uncooked_
0:100	−0.4	−7.6	−7.2
15:85	−5.5	−10.3	−4.8
30:70	−3.1	−8.3	−5.2
45:55	−2.2	−10.3	−8.1
55:45	−2.1	−13.4	−11.3
70:30	−1.9	−9.9	−8.0
85:15	−0.2	−8.0	−7.8
100:0	−0.3	−7.1	−6.9

Note: ∆ means the fractionation values of δ^2^H between the raw materials, uncooked noodles and cooked noodles.

**Table 3:** The mean ± standard deviation of δ^18^O in the raw materials, uncooked noodles and cooked noodles with different formulations of wheat gluten powder and starch powder.

Gluten–Starch Ratio [*w*/*w*]	Raw Materials	Uncooked Noodles	Cooked Noodles
0:100	29.1 ^cA^ ± 0.2	28.4 ^aAB^ ± 0.8	27.7 ^cB^ ± 0.2
15:85	29.6 ^bcA^ ± 0.2	28.9 ^aA^ ± 0.1	29.2 ^bA^ ± 0.4
30:70	30.6 ^abAB^ ± 0.8	29.3 ^aB^ ± 0.7	30.9 ^aA^ ± 0.3
45:55	30.4 ^aB^ ± 0.3	29.6 ^aC^ ± 0.3	31.3 ^aA^ ± 0.3
55:45	30.3 ^abcA^ ± 0.1	28.7 ^aB^ ± 0.4	30.1 ^abA^ ± 1.5
70:30	30.6 ^abcA^ ± 0.3	28.7 ^aC^ ± 0.4	29.9 ^abB^ ± 0.0
85:15	31.2 ^aA^ ± 0.2	28.8 ^aB^ ± 0.5	28.9 ^bcB^ ± 0.6
100:0	31.5 ^aA^ ± 0.3	28.9 ^aB^ ± 0.6	28.6 ^bcB^ ± 0.7

Note: in a–c, different mean values in the same column with superscripts of lowercase letters are significantly different (*p* < 0.05) according to Duncan’s multiple range test. In A–C, different mean values in the same line with different superscripts of uppercase letters are significantly different (*p* < 0.05) according to Duncan’s multiple range test.

**Table 4:** The **∆**δ^18^O value of the raw materials, uncooked noodles and cooked noodles with different formulations of wheat gluten powder and starch powder.

Gluten–Starch Ratio [*w*/*w*]	∆ δ^18^O _uncooked–raw_	∆ δ^18^O _cooked–raw_	∆ δ^18^O _cooked–uncooked_
0:100	−0.7	−1.4	−0.7
15:85	−0.7	−0.4	0.3
30:70	−1.2	0.3	1.6
45:55	−0.9	0.9	1.7
55:45	−1.7	−0.2	1.4
70:30	−1.9	−0.7	1.2
85:15	−2.4	−2.3	0.1
100:0	−2.5	−2.9	−0.3

Note: ∆ means the fractionation value of δ^2^H between the raw materials, uncooked noodles and cooked noodles.

**Table 5:** The combined analysis of variance for the stable hydrogen and oxygen isotopic ratios of the noodles.

Source of Variation			δ^2^H‰		δ^18^O‰
	df	MS	*p* Value	MS	*p* Value
Boiling Process	1	595.03 **	<0.001	11.49 **	<0.001
Formulation	7	1423.6 **	<0.001	4.38 **	<0.001
Boiling Process × Formulation	14	5.485	0.452	2.05 *	0.012
Error	48	5.39		1.064	

Note: (df) degree of freedom; (MS) mean square; ** highly statistically significant effect (*p* < 0.01); * statistically significant effect (*p* < 0.05).

## Data Availability

The original contributions presented in the study are included in the article, further inquiries can be directed to the corresponding authors.

## Appendix A

**Table A1:** Optimal cooking times for noodles with different gluten and starch formulations.

Gluten–Starch Ratio [*w*:*w*]	Cooking Time/Min
0:100	4.5
15:85	5
30:70	5.5
45:55	6
55:45	3
70:30	2
85:15	2
100:0	2
